# Aerobic phenol degradation using native bacterial consortium via ortho–and meta–cleavage pathways

**DOI:** 10.3389/fmicb.2024.1400033

**Published:** 2024-08-05

**Authors:** Sara Shebl, Doaa A. Ghareeb, Safaa M. Ali, Nevine Bahaa El Din Ghanem, Zakia A. Olama

**Affiliations:** ^1^Department of Botany & Microbiology, Faculty of Science, Alexandria University, Alexandria, Egypt; ^2^Department of Biochemistry, Faculty of Science, Alexandria University, Alexandria, Egypt; ^3^Nucleic Acid Research Center, City of Scientific Research and Technology Applications, Alexandria, Egypt

**Keywords:** aerobic phenol degrading genes, bacterial consortium, enzymes activities, gene expression, bioremediation

## Abstract

Effective bioremediation of a phenol-polluted environment harnesses microorganisms’ ability to utilize hazardous compounds as beneficial degraders. In the present study, a consortium consisting of 15 bacterial strains was utilized. The current study aims to monitor the phenol biodegradation pathway. The tested consortium showed effective potential in the bioremediation of phenol-contaminated industrial wastewater. The enzymatic studies conducted brought to light that the bacterial consortium under test was proficient in degrading phenol under aerobic conditions while exhibiting the simultaneous expression of both ortho- and meta-cleavage pathways. It was observed that pheA, pheB, and C12O genes were maximally expressed, and the enzymes responsible for phenol degradation, namely, phenol hydroxylase, catechol 1,2-dioxygenase, and catechol 2,3-dioxygenase, reached maximum activity after 48 h of incubation with a 20-ppm phenol concentration. To gain a deeper understanding of the activation of both ortho- and meta-cleavage pathways involved in phenol degradation, a technique known as differential display reverse transcriptase polymerase chain reaction (DDRT-PCR) was applied. This method allowed for the specific amplification and detection of genes responsible for phenol degradation. The expression levels of these genes determined the extent to which both ortho- and meta-cleavage pathways were activated in response to the presence of phenol.

## 1 Introduction

Environmental pollution is an unfortunate consequence of modern industrial society, leading to the presence of anthropogenic organic compounds in the environment. This poses a significant public health concern as soil, lakes, rivers, and seas are highly contaminated with various toxic compounds, including phenol, ammonia, cyanides, thiocyanate, phenol formaldehyde, acrylo- and aceto-nitrile, mercury, and heavy metals (Siddiqua et al., 2022). Phenol is a colorless crystalline solid with a distinct odor, possessing physico-chemical properties such as low volatility, high solubility in water, and the ability to dissolve in organic solvents (Martins et al., 2023). It enters ecosystems primarily through industrial activities, including the discharge of untreated wastewater and improper disposal of industrial waste (Ahmaruzzaman et al., 2024).

Phenol exhibits toxic effects on biota, including inhibition of enzyme activity, disruption of cell membranes, and interference with metabolic processes. Specific impacts on aquatic organisms include reduced growth, impaired reproduction, and even acute mortality. In Egypt, industrial sources of phenol include petrochemical refineries, chemical manufacturing plants, and certain agricultural practices that utilize phenolic compounds as pesticides or herbicides (Bhoi et al., 2024). The efficiency of phenol biodegradation is governed by numerous factors, such as the microorganisms involved in the degradation process, the enzymes responsible for the degradation, the degradation mechanism, and the environmental factors that may impact the process (Narayanan et al., 2023).

Biological treatment technologies have emerged as a promising alternative due to their low environmental impact, ability to produce no toxic end products, and their low cost. Many microorganisms have been found to be capable of metabolizing organic pollutants such as phenol, with metabolic processes being governed by specific enzymes for each type of reaction (Kumari and Das, 2023). Microbial consortia offer distinct advantages over individual microorganisms for phenol biodegradation (Lü et al., 2024). These consortia possess a diverse range of metabolic capabilities, ensuring a broader substrate utilization spectrum. Furthermore, microbial consortia can exhibit synergistic interactions that improve phenol degradation rates and increase resilience against environmental fluctuations (Huang et al., 2024).

There are several challenges associated with handling phenol by microbes. First, phenol is a recalcitrant compound, requiring the presence of specific enzymes, such as phenol hydroxylases, to initiate the degradation process (Kumari and Das, 2023). Second, phenol can inhibit microbial growth and activity at high concentrations (Lobiuc et al., 2023). In addition, the presence of other co-contaminants or environmental factors such as heavy metals or high salinity can complicate phenol degradation (Rashid et al., 2024). Finally, the degradation of phenol can be a slow process, requiring extended periods of time for complete removal (Bibi et al., 2023). Overall, the successful degradation of phenol by microbes necessitates overcoming these challenges.

During the process of phenol degradation, phenol hydroxylase initially transforms phenol into catechol. The subsequent cleavage of the catechol ring occurs via either the ortho-pathway, mediated by catechol 1,2-dioxygenase, or the meta-pathway, mediated by catechol 2,3-dioxygenase. These intermediates are then converted into non-toxic end products via the tricarboxylic acid cycle (Sun et al., 2022). Our findings indicate that the bacterial strains in the tested consortium utilize both the meta- and ortho-pathways for phenol degradation, as evidenced by the simultaneous activation of catechol 1,2-dioxygenase and catechol 2,3-dioxygenase.

Characterizing the enzymes involved in phenol biodegradation within microbial consortia is crucial for underlying mechanisms and optimizing degradation processes. Furthermore, assessing gene expression patterns provides valuable information on the transcriptional regulation of phenol degradation genes in microbial consortia. RT-PCR enables the quantification of gene expression levels under varying environmental conditions, facilitating the identification of key genes involved in phenol degradation pathways. This information is essential for understanding the regulatory networks that govern phenol biodegradation.

This study aimed to address the existing knowledge gap by justifying the use of microbial consortia, investigating associated enzymes, and assessing gene expression using RT-PCR for phenol biodegradation. By elucidating the rationale behind these approaches, we hope to provide a foundation for the development of effective and sustainable phenol remediation strategies. Ultimately, this research has the potential to contribute to the broader field of environmental biotechnology and advance our understanding of microbial processes for the remediation of hazardous pollutants.

## 2 Materials and methods

### 2.1 Wastewater samples

Wastewater effluents containing phenol were collected from Alexandria Mineral Oils Co. (‘AMOC’), an operational facility in Egypt that has been in continuous production for several years. The effluent was weekly analyzed, and the phenol concentration was estimated along with other parameters as illustrated in Table 1.

**Table 1:** Industrial effluent characterization.

Parameter	Value (mg/l)
pH	6–9
BOD	150–250
COD	300–600
Phenol	14–20
Benzene	1–100
Benzo(a)pyrene	1–100
Oil & Grease	100–300
Chrome	0.1–100
Lead	0.2–10

### 2.2 Microorganisms

Microorganisms used in the present study were 15 bacterial phenol degraders isolated from oil refinery wastewater and were identified using 16S rRNA. All the bacterial strains were maintained on Mueller Hinton Agar at 35°C with monthly transfer on fresh media.

### 2.3 Isolation, purification, and identification of phenol degrader bacteria

The pour-plate method was used to isolate phenol-degrading bacteria on P-NA using enrichment media containing phenol, P-NB, and P-NA (Usman et al., 2021). Only the single colonies grown on the P-NA medium were transferred independently to a fresh medium in a Petri plate and subcultured in several slants of maintenance medium (Mueller Hinton Agar), labeled with the initial designations, and stored for future investigation.

Morphological examinations and biochemical analysis using the API 20NE kit (bioMerieux, Marcy Etoile, France) have been evaluated for the identification of phenol degraders. Confirmation of the identification was evaluated using 16S rDNA sequencing. Genomic DNA was extracted according to Hassen et al. (2018) from an overnight-grown culture, and PCR amplification was performed using a forward primer (5′-AGAGTTTGATCMTGGCTCAG-3′) and a reverse primer (3′-TACCTTG TTACGACTT-5′) for 16S rRNA. The obtained 16S rRNA sequences were integrated into the database using the automatic alignment tool, and a phylogenetic tree was generated through distance matrix analysis using the NT system. Database searches and comparisons were performed using the BLAST search tool with the National Centre for Biotechnology Information (NCBI) database, and accession numbers were obtained.

### 2.4 Inoculum preparation and standardization

The McFarland standards serve as a benchmark for regulating the cloudiness of bacterial suspensions, ensuring that the bacterial count falls within a predetermined range for consistent microbial testing. These initial standards were created through the precise combination of specified quantities of barium chloride and sulfuric acid. When these two compounds are mixed, they produce a precipitate of barium sulfate, leading to the development of turbidity in the solution. A 0.5 McFarland standard is prepared by mixing 0.05 mL of 1.175% barium chloride (BaCl2•2H2O), with 9.95 mL of 1% sulfuric acid (H2SO4).

The standard can be compared visually to a suspension of bacteria in sterile saline or nutrient broth from a freshly prepared culture of each isolate (16–24-h-old cultures), and 4 to 5 colonies were emulsified in 5 mL saline solution (0.85%) to achieve a turbid suspension. Matching the turbidity with the unaided eye by holding the bacterial suspension and McFarland tubes side by side and viewing them against a black-lined background (Ghanem and Olama, 2014). If the bacterial suspension is too turbid, it can be diluted with more medium. If the suspension lacks sufficient turbidity, additional bacteria can be introduced. A 0.5 McFarland standard yields an optical density that is equivalent to the density of a bacterial suspension containing 1.5 ×10-6 CFU/mL (Pervez et al., 2014).

### 2.5 Growth kinetics

Both phenol degradation and the optical density of the bacterial consortium were monitored over a period of 3 days every 6 h. The bacterial consortium was allowed to grow on both a minimal medium fortified with 20 ppm phenol (MM) and a minimal medium without phenol (C). Phenol concentration was evaluated by spectrophotometric analysis using the HACH^®^ phenol kit in accordance with the manufacturer’s instructions (Setlhare et al., 2021). The concentration was reported in mg/l. Meanwhile, the growth of the bacterial consortium was monitored in both media using a UV–Vis spectrophotometer at a wavelength of 600 nanometers (λ600) (Yavari-Bafghi et al., 2023).

### 2.6 Fermentation process

#### 2.6.1 Cultivation and preparation of cell-free extract

The isolated bacterial strains were enriched in nutrient broth and then cultivated on a minimal medium containing g/l: KH2PO4, 0.07; K2HPO4, 0.125; FeCl3, 0.07; and CaCl2, 0.003, once without phenol as a control and the other one fortified with 20 ppm phenol as a sole carbon and energy source (Kotoky and Pandey, 2021). After 48 h of incubation at 35°C under shaken conditions, the cells were harvested from both media by centrifugation (10,000 rpm, for 10 min at 5°C). The pellets were washed twice with phosphate buffer (50 mM, pH 7.0) and re-suspended in 10 mL of the same buffer. Suspensions were sonicated with repeated 40 s bursts alternated with 1 min cooling on ice. The cellular debris was subjected to centrifugation at 12,000 rpm for a duration of 15 min. The supernatants (cell-free extract) were used for crude enzyme assays (Schmidt, 2013), although total protein estimation was evaluated using the Biuret method with a commercial kit.

#### 2.6.2 Enzyme activity

Bacterial enzymes responsible for phenol degradation, namely, phenol hydroxylase, catechol 1,2-dioxygenase, and catechol 2,3-dioxygenase, were assessed. Enzyme activities were evaluated for each bacterial strain separately using a cell-free extract of the corresponding bacterial culture, one on a minimal medium without phenol as a control and the other on a minimal medium with phenol, one at a time. Specific enzyme activity was calculated as enzyme units per 1 mg protein (IU/mg protein).

##### 2.6.2.1 Phenol hydroxylase activity

Phenol hydroxylase activity was estimated using a reaction mixture of cell-free extract, 0.02 mg; Tris–HCl (pH 8.0), 50 mM; and phenol, 200 mg/L. The reaction was started with the addition of 2.5 μM NADH to the reaction mixture containing fresh crude extracts. Phenol hydroxylase activity was measured in correspondence to NADH consumption at a wavelength of 340 nm using a UV spectrophotometer. One unit of activity was defined as the amount of enzyme that oxidized 1 μM NADH per min in the presence of phenol (Shebl et al., 2022).

##### 2.6.2.2 Catechol 1,2-dioxygenase activity

Catechol 1,2-dioxygenase activity was detected in the cell-free extracts using a reaction mixture of 1 mL cell-free extract, 1 mL of 0.8 mM catechol, 0.8 mL of 50 mM Tris–HCl buffer solution (pH 8.0), and 0.2 mL of 0.1 mM 2-mercaptoetanol. The reaction mixture was incubated at 30°C in a water bath for 10 min before analysis. The enzyme activity was measured using a UV spectrophotometer at a wavelength of 260 nm in correspondence to the formation of cis, cis-muconic acid as described in a previous study (Mollasalehi and Shajari, 2021).

##### 2.6.2.3 Catechol 2,3-dioxygenase activity

The activity of catechol 2,3-dioxygenase was assayed in the cell-free extract using a reaction mixture of 1 mL of cell-free extract, 0.8 mL of 50 mM phosphate buffer (pH 7.0), and 1 mL of 0.3 mM catechol. The reaction mixture was incubated at 30°C in a water bath for 10 min before analysis. The enzyme activity was measured using a UV spectrophotometer at a wavelength of 375 nm as described in a previous study (Mollasalehi and Shajari, 2021).

#### 2.6.3 Statistical analysis

All experiments were performed in triplicate. The results obtained in the present study were analyzed through multiple comparison ANOVA tests using IBM SPSS Statistics.

### 2.7 Phenol degradation assessment via molecular approach

#### 2.7.1 Differential-display reverse transcription-PCR (DDRT-PCR)

##### 2.7.1.1 RNA extraction

The bacterial consortium was allowed to grow on a minimal medium without phenol as a control and on a minimal medium fortified with 20 ppm phenol, and pellets were collected over a period of 3 days every 6 h. After incubation, cultures were centrifuged, and pellets were frozen and transferred to an RNase-free tube. The RNA extraction procedure was conducted by employing the GENEzol TriRNA Pure Kit (Geneaid Biotech) as per the instructions provided by the manufacturer (Baker et al., 2023).

##### 2.7.1.2 DDRT-PCR

A High-Capacity cDNA Reverse Transcription Kit (Invitrogen) was used for the generation of the first cDNA strand from extracted total RNA following the manufacturer’s instructions. A total of 12 primers (specific for phenol degradation genes) were subjected to PCR for cDNA amplification. The resulting patterns were analyzed using statistical methods to determine the molecular weight of different bands that appeared on the agarose gel. The PCR reaction was performed for 4 min at 95°C followed by 40 cycles each of 40 s at 94°C, 50 s at 30°C, and 50 s at 72°C, followed by a supplementary 10 min at 72°C. After amplification by PCR, the products were checked by 2% agarose gel electrophoresis. Bands with molecular weights of 350, 450, 500, 575, 600, and 800 bp were analyzed using a purification gel extraction kit and then sequenced using the specified primer (which was initially used for amplification). Sequence data were analyzed after the conversion of nucleotide to amino acid sequences using different bioinformatic programs (CLC and BioEdit), and the final data were compared to the gene bank data (Rather et al., 2023).

##### 2.7.1.3 Phenol-degrading genes

In a trial to analyze the gene expression patterns of the cells and gain insights into the molecular mechanisms underlying phenol biodegradation pathways, mRNA was extracted from cells cultured on phenol at different growth stages, and the reverse transcription polymerase chain reaction (RT-PCR) was evaluated. Specific primers were utilized to examine the key functional genes responsible for phenol degradation in the bacterial consortium, namely, phenol hydroxylase large subunit (encoded by *pheA*), phenol hydroxylase small subunit (encoded by *pheB*), catechol 1,2-dioxygenase (encoded by *C12O*), catechol 2,3-dioxygenase (encoded by *C23O*), muconate cycloisomerase (encoded by *catB*), and 2-hydroxymuconic semi aldehyde dehydrogenase (encoded by *HMSD*) as illustrated in Table 2. The primers were designed based on the known sequence of respective genes in the NCBI GenBank.^1^

**Table 2:** Phenol-degrading genes and their primers.

Genes	Enzyme	Primers	Bp	References
*pheA*	Phenol hydroxylase	CCAGG(C/G) (C/G/T) GA(G/A) AA(A/G)GAGA(A/G)GAA(G/A)CT	450	Tuan et al. (2011)
		CGG(A/T) A(G/A)CCGCGCCAGA ACCA		
*pheB*	Phenylphosphate carboxylase beta subunit	TGACCATGGCCGTTTCCTAC	500	Saa et al. (2010)
		TCTTGACCATTTCGGGGTCG		
*C12O*	Catechol 1,2-Dioxygenase	AGACCTGGAAATCACCGAAGACG	610	Cámara et al. (2007)
		GGGTGGCGTGGCAAAGTCGTC		
*C23O*	Catechol 2,3-dioxygenase	GARCTSTAYGCSGAYAAGGAR	350	Tahya et al. (2019)
		RCCGCTSGGRTCGAAGAARTA		
*catB*	muconate cycloisomerase	ATGACCATTTCTCAGGCGCA	800	Saa et al. (2010)
		GTCGCGCTTCATCATTTCCC		
*HMSD*	2-hydroxymuconic semialdehyde dehydrogenase	CGCCAGAACCACTTGTCRRTCCA	575	Marín et al. (2012)
		ACCGGGATATTTYTC TTCSAGCA		

## 3 Results

### 3.1 Isolation and purification of phenol degraders

A total of 15 bacterial phenol degraders were isolated from oil refinery wastewater and selected for further investigation. Mixed bacterial strains (consortium) were more efficient degraders than single strains.

### 3.2 Phenol degrader bacteria identification

The bacterial isolates were identified using 16S rDNA sequencing after DNA extraction, with a purity level in the range of 1.79–1.9. The sequences obtained are compared to the nucleotide sequences of the international database, and the molecular analysis of the different bacterial isolates revealed that Strain 1 was identified as *Pseudomonas aeruginosa* I with 100% similarity, while the others were identified as *Klebsiella pneumonia* I*, Bacillus cereus, Pseudomonas monteilii, Bacillus subtilis, Pseudomonas mosselii, Staphylococcus equorum, Bacillus benzoevorans, Bacillus circulans, Pseudomonas fulva, Pseudomonas aeruginosa* II*, Pseudomonas putida, Burkholderia cepacia, Bacillus cereus, and Klebsiella pneumonia* II with 97.33, 99.35, 98.4, 97.9, 98, 99.7, 97.9, 97.8, 96.2, 97.3, 99, 97.25, 99.6, and 96.4% similarity, respectively (Table 3). Database search and comparisons were evaluated with the BLAST search using the National Centre for Biotechnology Information (NCBI) database. The 16S rRNA sequence was deposited in the NCBI GenBank nucleotide sequence database under certain accession numbers (Table 3).

**Table 3:** Bacterial identification data.

Strain number	Identification	Identified accession number	Identity %
1	*Pseudomonas aeruginosa*	*MW598285*	100
2	*Klebsiella pneumoniae Ι*	*MW585395*	97.3
3	*Bacillus cereus*	*MW585396*	99.4
4	*Pseudomonas monteilii*	*MW585595*	98.4
5	*Bacillus subtilis*	*MW585596*	97.9
6	*Pseudomonas mosselii*	*MW585691*	98.0
7	*Staphylococcus equorum*	*MW585694*	99.7
8	*Bacillus benzoevorans*	*MW597321*	97.9
9	*Bacillus circulans*	*MW597408*	97.8
10	*Pseudomonas fulva*	*MW598162*	96.2
11	*Pseudomonas aeruginosa*	*MW598228*	97.3
12	*Pseudomonas putida*	*MW598278*	99.0
13	*Burkholderia cepacia*	*MW579472*	97.3
14	*Bacillus cereus*	*MW598367*	99.6
15	*Klebsiella pneumoniae ΙΙ*	*MW598404*	96.4

### 3.3 Growth kinetics

**Figure 1:**
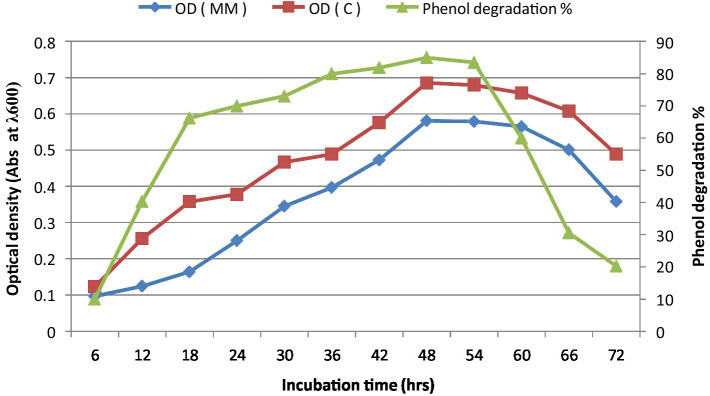
Growth kinetics with phenol degradation percentage at different time intervals. ^*^C is minimal medium without phenol (Control), MM is minimal medium with phenol (Test), and OD is optical density.

The bacterial consortium growth was monitored during phenol degradation. Maximum degradation was reported after 48 h of incubation when grown on MM medium, with the maximum biomass production observed between 48 and 54 h of incubation (Figure 1).

### 3.4 Phenol-degrading enzyme activity

In a trial to test the mechanism of phenol degradation using the 15 tested bacterial strains (Table 3) one at a time, the activities of phenol-degrading enzymes, specifically phenol hydroxylase, catechol 1,2-dioxygenase, and catechol 2,3-dioxygenase, were measured. The results in Tables 4–6 revealed that the three tested enzymes reported high activities with all bacterial strains under investigation using a minimal medium fortified with phenol.

**Table 4:** Phenol hydroxylase activity as assessed by the tested bacterial strains.

Phenol hydroxylase activity (IU/mg protein)			Fold increase
Bacterial strain	Minimal medium without phenol	Minimal medium with phenol	
*Pseudomonas aeruginosa*	0.541	1.380	2.55 ± 0.61C
*Klebsiella pneumoniae Ι*	0.338	1.358	4.02 ± 0.92B
*Bacillus cereus*	0.299	1.322	4.42 ± 0.88B
*Pseudomonas monteilii*	0.230	1.397	6.07 ± 1.52A
*Bacillus subtilis*	0.310	1.750	7.42 ± 1.63A
*Pseudomonas mosselii*	0.417	2.050	4.92 ± 1.03B
*Staphylococcus equorum*	0.250	1.843	7.38 ± 1.69A
*Bacillus benzoevorans*	0.221	1.640	6.13 ± 1.28A
*Bacillus circulans*	0.403	1.494	3.71 ± 0.66B
*Pseudomonas fulva*	0.296	1.402	4.73 ± 0.94B
*Pseudomonas aeruginosa*	0.637	2.568	4.03 ± 0.81B
*Pseudomonas putida*	0.198	1.315	7.66 ± 1.83A
*Burkholderia cepacia*	0.551	1.527	2.77 ± 0.47C
*Bacillus cereus*	0.326	1.537	4.72 ± 0.94B
*Klebsiella pneumoniae ΙΙ*	0.124	1.224	9.84 ± 2.46A

^*^A is the most significant, B is insignificant, and C is the least significant.

**Table 5:** Catechol 1, 2-dioxygenase activity as assessed by the tested bacterial strains.

Catechol 1,2-dioxygenase activity (nmol/ min/mg)			Fold increase
Bacterial strain	Minimal medium without phenol	Minimal medium with phenol	
*Pseudomonas aeruginosa*	19.20	46.60	2.43 ± 0.58B
*Klebsiella pneumoniae Ι*	17.37	40.69	2.34 ± 0.54B
*Bacillus cereus*	14.29	40.09	2.81 ± 0.71A
*Pseudomonas monteilii*	16.99	43.27	2.55 ± 0.61A
*Bacillus subtilis*	13.04	36.65	2.72 ± 0.68A
*Pseudomonas mosselii*	15.65	44.03	3.78 ± 0.91A
*Staphylococcus equorum*	17.04	37.15	2.18 ± 0.01C
*Bacillus benzoevorans*	19.17	47.09	2.46 ± 0.51B
*Bacillus circulans*	21.29	43.06	2.02 ± 0.35C
*Pseudomonas fulva*	21.23	36.71	1.73 ± 0.25C
*Pseudomonas aeruginosa*	38.21	53.65	1.40 ± 0.224C
*Pseudomonas putida*	13.44	37.30	3.21 ± 0.79A
*Burkholderia cepacia*	15.92	40.74	2.56 ± 0.62A
*Bacillus cereus*	16.69	37.06	2.22 ± 0.41B
*Klebsiella pneumoniae ΙΙ*	17.29	34.15	1.97 ± 0.53C

^*^A is the most significant, B is insignificant, and C is the least significant.

**Table 6:** Catechol 2, 3-dioxygenase activity as assessed by the tested bacterial strains.

Catechol 2,3-dioxygenase activity (IU/mg protein)			Fold increase
Bacterial strain	Minimal medium without phenol	Minimal medium with phenol	
*Pseudomonas aeruginosa*	3.980	4.685	1.18 ± 0.24C
*Klebsiella pneumoniae Ι*	4.081	4.820	1.42 ± 0.32A
*Bacillus cereus*	4.058	4.783	1.18 ± 0.17C
*Pseudomonas monteilii*	3.914	4.649	1.19 ± 0.21C
*Bacillus subtilis*	4.313	6.129	1.05 ± 0.16C
*Pseudomonas mosselii*	4.139	4.962	1.20 ± 0.24B
*Staphylococcus equorum*	4.204	5.965	1.72 ± 0.37A
*Bacillus benzoevorans*	4.159	5.513	1.33 ± 0.28A
*Bacillus circulans*	4.177	5.575	1.33 ± 0.28A
*Pseudomonas fulva*	3.589	4.483	1.25 ± 0.24B
*Pseudomonas aeruginosa*	4.389	6.239	2.24 ± 0.54A
*Pseudomonas putida*	3.938	4.652	2.85 ± 0.68A
*Burkholderia cepacia*	4.150	5.265	1.27 ± 0.22B
*Bacillus cereus*	3.693	4.526	1.23 ± 0.23B
*Klebsiella pneumoniae ΙΙ*	3.771	4.547	1.21 ± 0.23B

^*^A is the most significant, B is insignificant, and C is the least significant.

Phenol hydroxylase exhibited the highest activity in all the bacterial strains, with approximately a 5.4-fold increase in activity when grown in the presence of phenol; on the contrary, catechol 1,2-dioxygenase and catechol 2,3-dioxygenase showed approximately a 2.4- and 1.4-fold increase, respectively.

Phenol hydroxylase activity was highly displayed with *Klebsiella pneumoniae ΙΙ, Pseudomonas putida,* and *Bacillus subtilis,* respectively, which exhibited 9.84-, 7.66-, and 7.42-fold increases (Table 4). In the case of Catechol 1, 2-dioxygenase, enzymes *Pseudomonas mosselii, Pseudomonas putida,* and *Bacillus cereus,* respectively, showed 3.78-, 3.21-, and 2.81-fold increases in enzyme activity, while the other tested strains showed lower activities (Table 5). Catechol 2,3-dioxygenase of *Pseudomonas putida, Pseudomonas aeruginosa,* and *Staphylococcus equorum* reported high activity with a 2.85-, 2.24-, and 1.72-fold increase in comparison with the other tested bacterial strains (Table 6).

**Figure 2:**
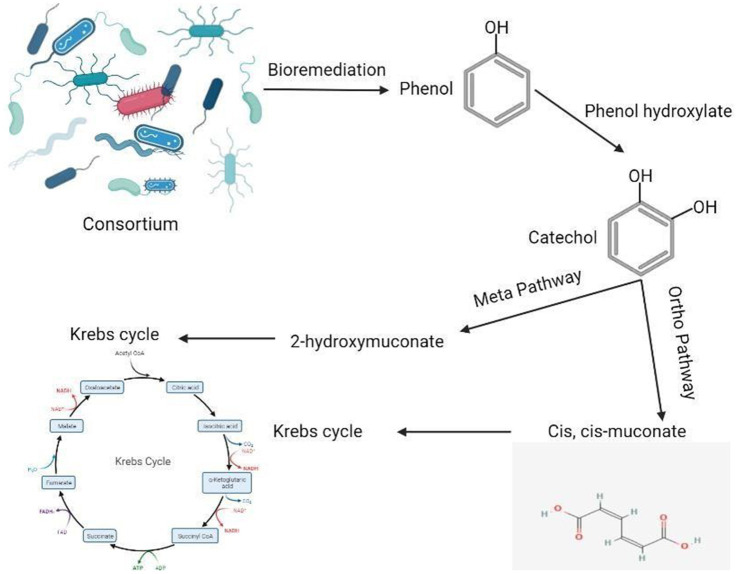
Phenol biodegradation pathway.

**Figure 3:**
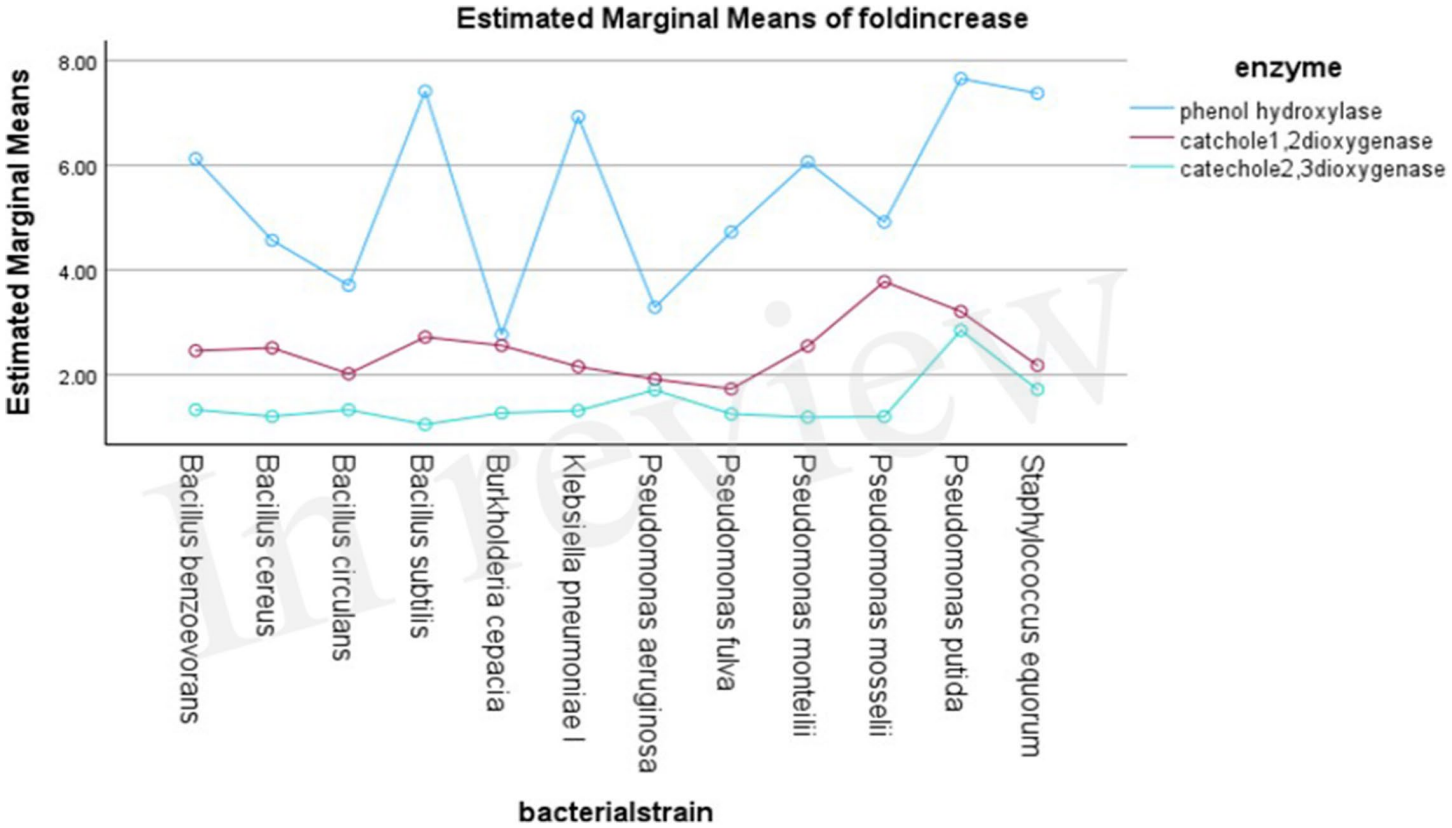
Trends in enzyme activity.

In the first step of the aerobic biodegradation of phenol, the phenol hydroxylase enzyme utilizes molecular oxygen for the addition of a newly second hydroxyl group in the ortho-position in the presence of a reduced pyridine nucleotide (NADHz) (Wilkes et al., 2023). The resulting catechol (l,2-dihydroxybenzene) molecule can then be degraded via ortho- and meta-cleavage pathways. In the ortho-pathway, catechol hydroxyl groups of the aromatic ring are cleaved by catechol 1,2-dioxygenase (intradiol fission), resulting in the production of cis, cis-muconic acid. This acid is further metabolized into Krebs cycle intermediates, while in the meta-pathway, ring fission occurs adjacent to the two hydroxyl groups of catechol (extradiol fission) (Mohd, 2022). The enzyme catechol 2,3-dioxygenase transforms catechol into 2-hydroxymuconic semialdehyde, which is further metabolized into intermediates of the Krebs cycle, as illustrated in Figure 2. Moreover, the results demonstrated the versatility of the bacterial strains under test in utilizing both ortho- and meta-cleavage pathways for phenol degradation. Notably, *Pseudomonas putida* was found to be a highly active organism, exhibiting robust enzyme expression. The results were analyzed using a multivariable ANOVA test, and it was confirmed that phenol hydroxylase activity showed the highest significance in the test (medium with phenol) compared to control trials (medium without phenol) as illustrated in Tables 4–6, while catechol 1, 2-dioxygenase and catechol 2,3-dioxygenase showed almost the same trend in enzyme activity (Figure 3).

### 3.5 Phenol degradation assessment via molecular approach

**Figure 4:**
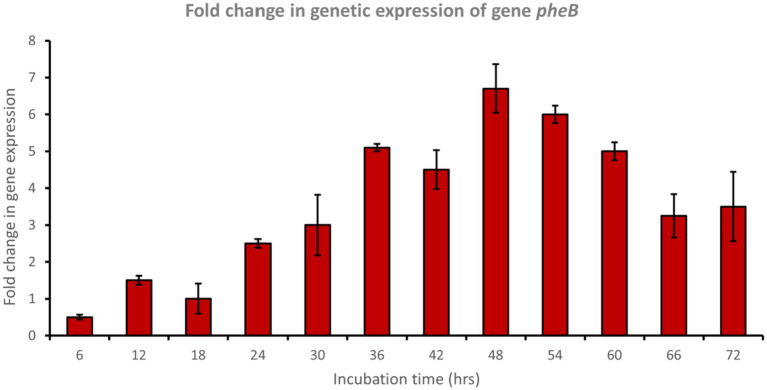
Fold change in gene expression of gene *pheA* by the bacterial consortium using MM medium fortified with phenol at different time intervals.

**Figure 5:**
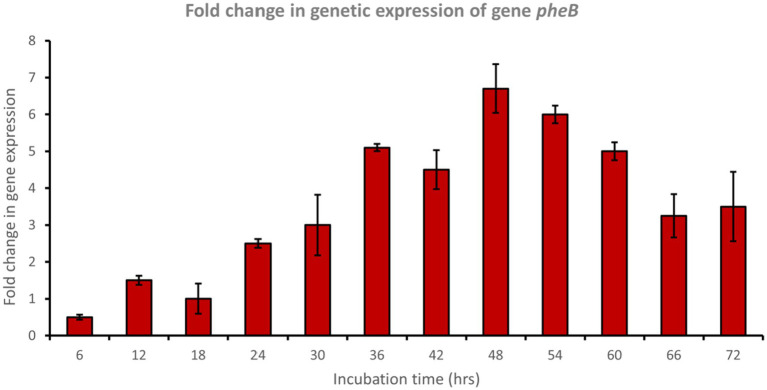
Fold change in gene expression of gene *pheB* by the bacterial consortium using MM medium fortified with phenol at different time intervals.

In a trial to study the phenol degradation pathways, the detection of the key functional genes in the biochemical pathway was performed via PCR using specific primers. Reverse transcription polymerase chain reaction (RT-PCR) experiments were evaluated using mRNA extracted from cells grown on phenol. The results confirmed the expression of *pheA* and *pheB* genes, which are known to encode for the phenol hydroxylase enzyme (large and small subunits, respectively), which plays a crucial role in the first step in the aerobic degradation pathway, which involves the hydroxylation of phenol to form catechol. Fold changes in genetic expression revealed that genes *pheA* and *pheB* were upregulated by approximately 8.5- and 6.5-fold, respectively, after 48 h of incubation with phenol (Figures 4, 5).

**Figure 6:**
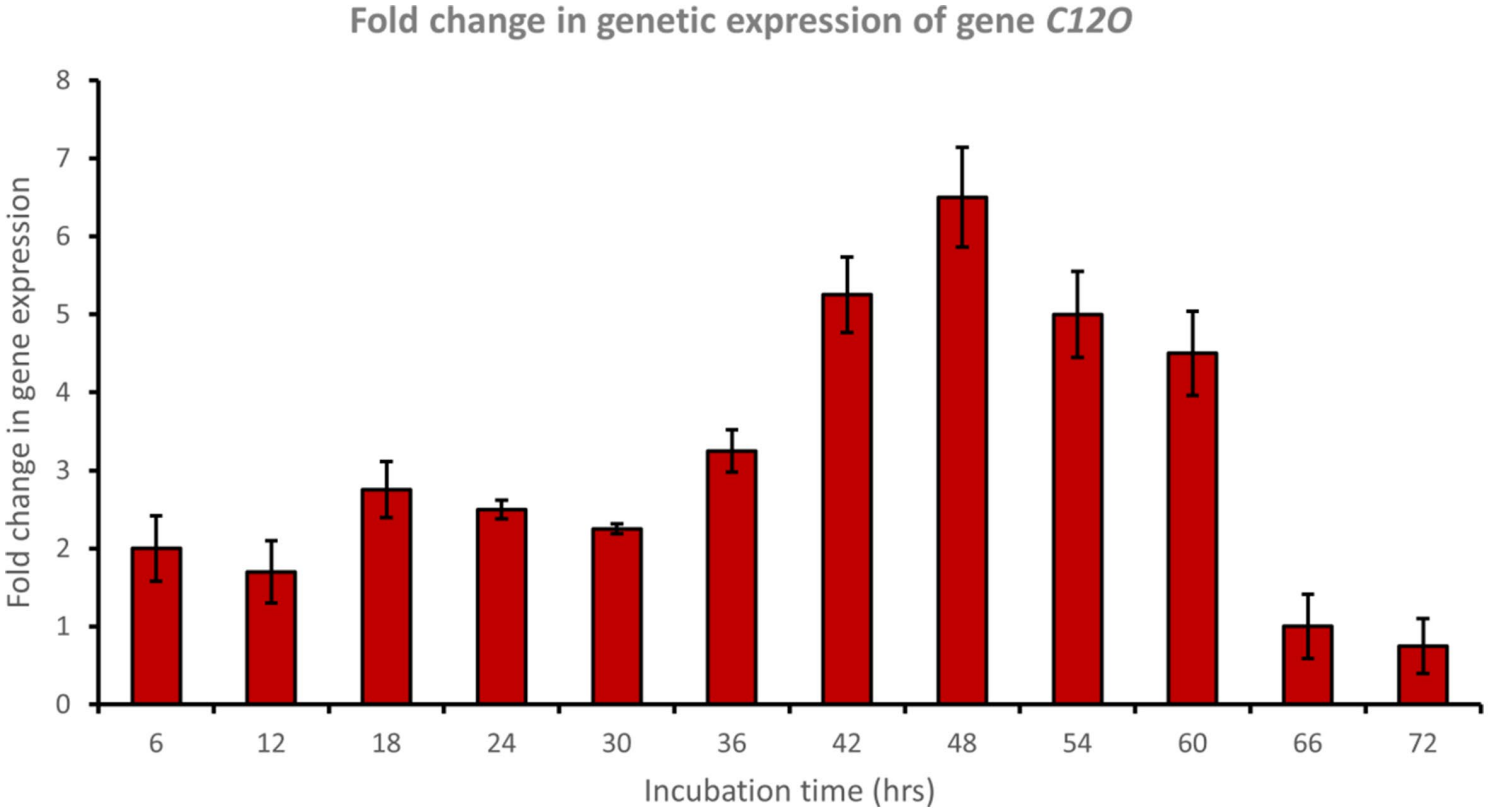
Fold change in gene expression of gene *C12O* by the bacterial consortium using MM medium fortified with phenol at different time intervals.

**Figure 7:**
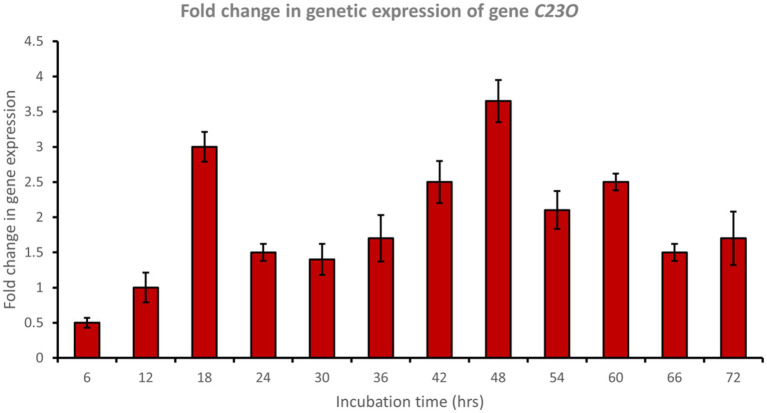
Fold change in gene expression of gene *C23O* by the bacterial consortium using MM medium fortified with phenol at different time intervals.

Catechol 1,2-dioxygenase (encoded by *C12O*) was upregulated approximately 6.5-fold after 48 h of incubation with phenol (Figure 6), while catechol 2,3-dioxygenase (encoded by *C23O*) was upregulated approximately 3.7-fold after 48 h of incubation with phenol (Figure 7). Cat genes play a crucial role in the initial ring cleavage of catechol, and their expression is often modulated by the presence of catechol.

**Figure 8:**
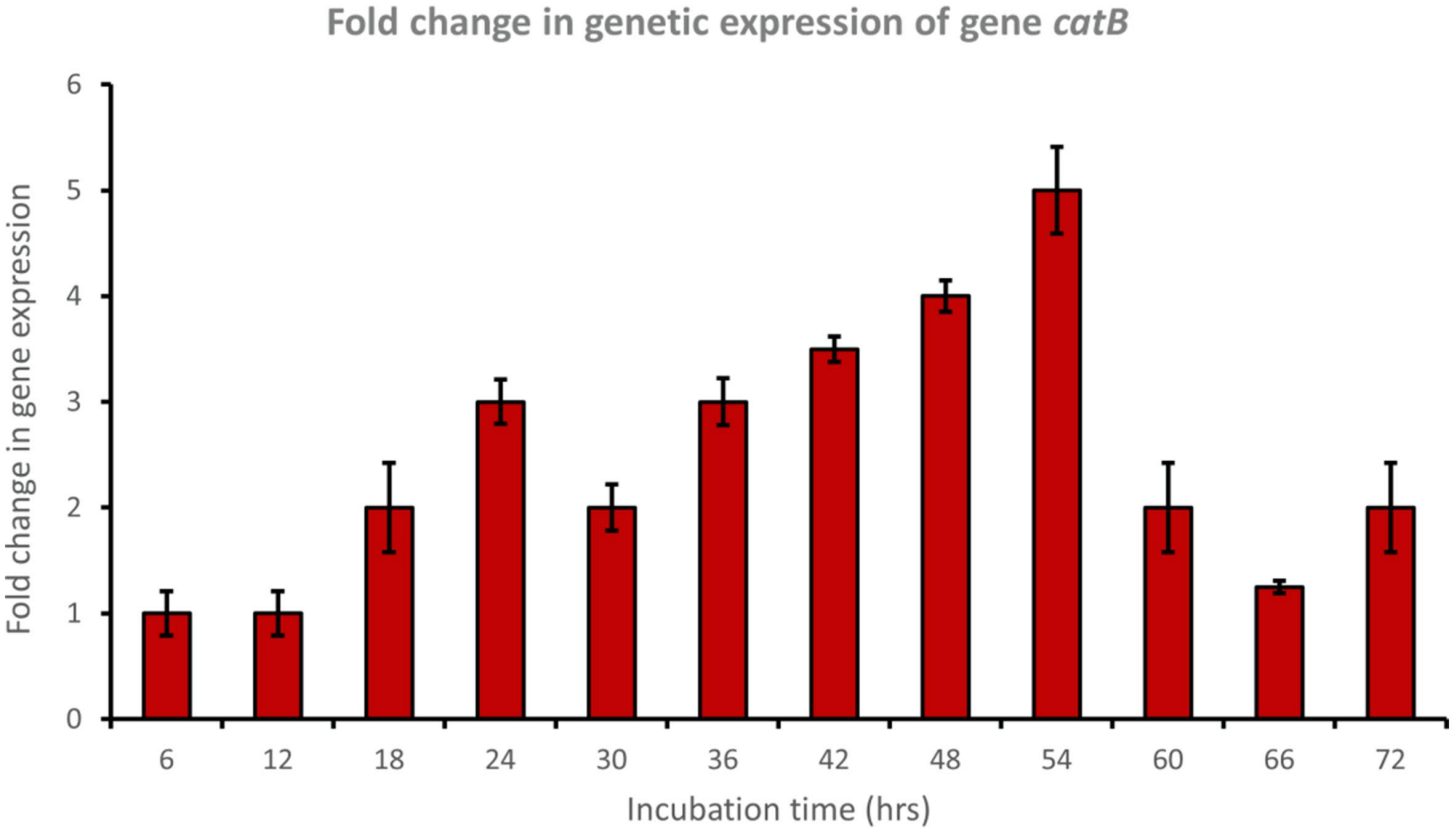
Fold change in gene expression of gene *catB* by the bacterial consortium using MM medium fortified with phenol at different time intervals.

**Figure 9:**
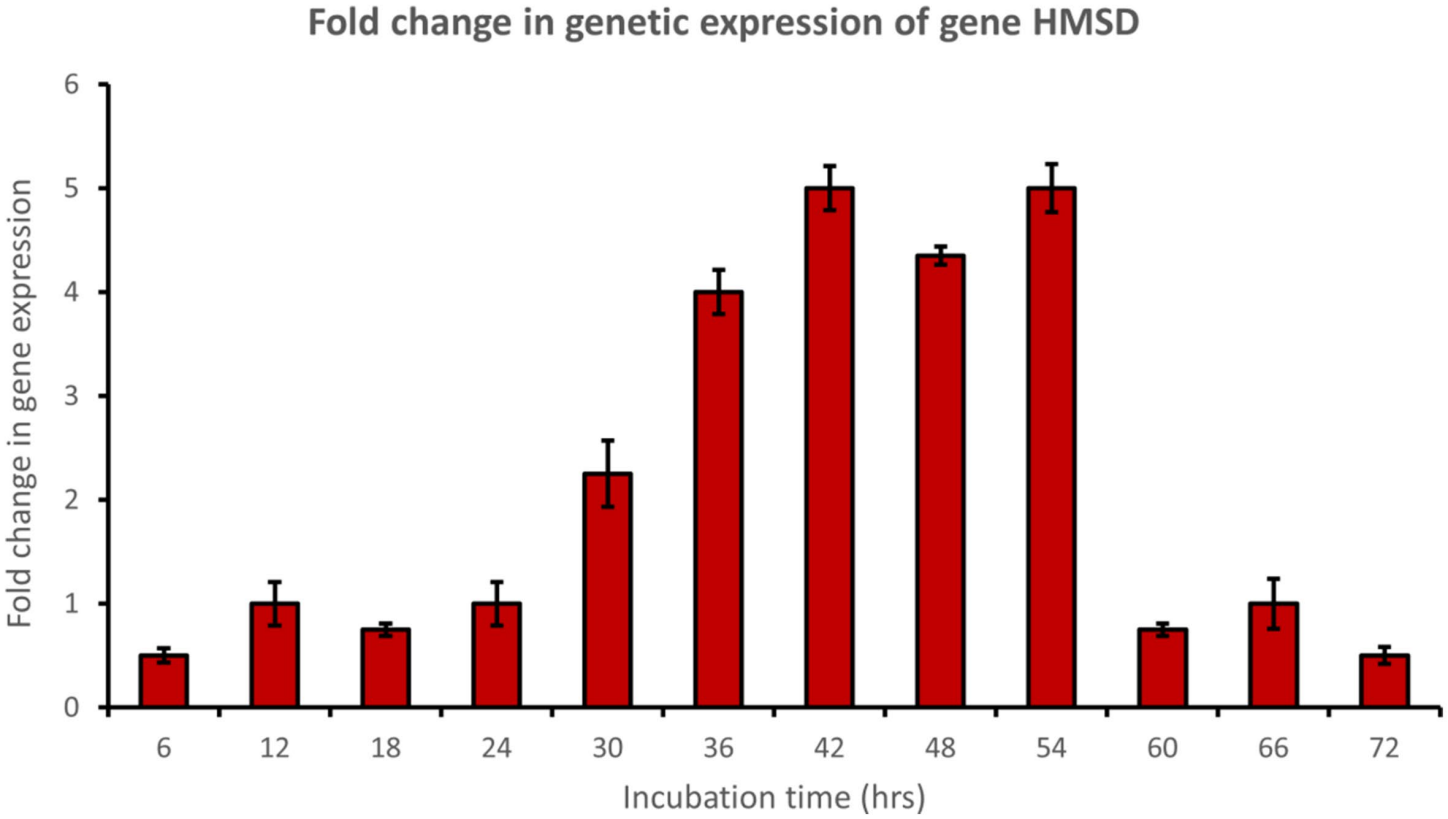
Fold change in gene expression of gene *HMSD* by the bacterial consortium using MM medium fortified with phenol at different time intervals.

Muconate cycloisomerase (*catB*) was upregulated approximately 5-fold after 54 h of incubation, indicating ortho-cleavage pathway activation by the bacterial consortium during phenol degradation (Figure 8). In addition, 2-hydroxymuconic semialdehyde dehydrogenase (*HMSD*) was upregulated approximately 5-fold during 42 to 54 h of incubation, indicating meta-cleavage pathway activation by the bacterial consortium during phenol degradation (Figure 9).

Therefore, a consortium is important for phenol elimination in wastewater. The consortium had the most important phenol degradation genes (all the tested genes): phenol hydroxylase, catechol 1,2-dioxygenase, catechol 2,3-dioxygenase, muconate cycloisomerase, and 2-hydroxymuconic semialdehyde dehydrogenase. These findings provide additional evidence for the activation of both ortho- and meta-cleavage pathways during phenol degradation by the bacterial consortium under investigation, which aligns with the results obtained from the tested enzymatic activities. The upregulation (expression) of *pheA* and *pheB* genes confirms the consortium’s ability to degrade phenol and the activation of the key enzyme phenol hydroxylase. In addition, the upregulation (expression) of *C12O* and *catB* genes indicates the activation of the ortho-cleavage pathway, while the *C23O* and *HMSD* genes suggest the activation of the meta-cleavage pathway.

## 4 Discussion

The efficiency of phenol biodegradation is governed by numerous factors, such as the microorganisms involved in the degradation process and the enzymes responsible for the degradation. The rationale for the use of mixed culture populations is that the microbial consortia can perform more complicated tasks and endure more changeable environments than monocultures (Shong et al., 2012). Since the three tested enzymes (phenol hydroxylase, catechol 1,2-dioxygenase, and catechol 2,3-dioxygenase) reported high activities with all bacterial strains under investigation using a minimal medium fortified with phenol, therefore phenol is a key inducer for all enzymes under investigation (Ehrt et al., 1995). Notably, *Pseudomonas putida* was found to be a highly active organism, exhibiting robust enzyme expression. The present findings are consistent with Cao et al. (2008) who reported that *Pseudomonas putida* has a high affinity for activating both the ortho- and meta-cleavage pathways during phenol degradation.

In addition, the results confirmed the expression of *pheA* and *pheB* genes, which involves the hydroxylation of phenol to form catechol (Harzallah et al., 2023). Elken et al. (2020) constructed a new PHE plasmid using *Pseudomonas* in a phenol-polluted environment and revealed that this plasmid comprises *pheA* and *pheB* genes as they play a crucial role in the first step of phenol biodegradation.

Medić and Karadžić (2022) reported activation of catechol 1, 2-dioxygenase in *Pseudomonas* during environmental bioremediation of hydrocarbons and phenolic compounds. The primary focus of Meier et al. (2022) is the characterization of catechol 1,2-dioxygenase (Acdo1p) and the examination of its role in *B. raffinosifermentans*, particularly regarding the degradation of catechol within tannic acid. Sasi and Suchithra (2023) reported upregulation of catechol 2,3-dioxygenase along with 2-hydroxymuconic semialdehyde dehydrogenase during phenol degradation using a new strain of *Bacillus cereus*, STV1713, isolated from petroleum-contaminated soil. Canul-Chan et al. (2023) proved the activation of catechol 2,3-dioxygenase (*C23O*) during the biological degradation of aromatic compounds using a native bacterial consortium with upregulation by 285.13 ± 29.39 pmol min/mg protein during the first 2 days of incubation, while Cat genes are involved in phenol degradation by *consortium* via ortho- and meta-aerobic pathways (Mohlam et al., 2018).

Ma et al. (2023) showed efficient aerobic catechol degradation using a bacterial consortium of *Rhodococcus*, *Pseudomonas, Pseudoclavibacter*, and *Raineyella,* indicating that degradation was achieved through the catechol ortho-cleavage pathway with upregulation of the muconate cycloisomerase enzyme Wolf et al. (2021) studied the phenol meta-degradation pathway in *S. solfataricus,* using phenol as the sole carbon source and activating the 2-hydroxymuconic semialdehyde dehydrogenase enzyme.

A recent study by Zhang et al. (2023) indicated that activation of both pathways coexists in the strain *Cupriavidus nantongensis* X1 for phenol aerobic biodegradation, ortho- and meta-pathways. C12O and C23O served as pivotal enzymes within two metabolic pathways. The degradation activities of enzyme *C23O* were 188-fold higher than those of *C12O in vitro*, which indicated that the meta-pathway was more efficient than the ortho-pathway for catechol degradation in strain X1.

The significant importance of the mixed culture as a bioremediation tool relies on previously reported studies suggesting that microbial consortia can utilize both meta- and ortho-pathways for the degradation of phenol, leading to more efficient degradation compared to the use of individual bacterial strains (Bai et al., 2021). Thus, the bacterial consortium under test effectively activates both pathways while degrading phenol, further supporting the results obtained from the enzyme activities and elucidating other findings in previous studies (Cao et al., 2008; Bai et al., 2021; Zhang et al., 2023).

## 5 Conclusion

The data from the present study concluded that:

- The use of a consortium consisting of 15 bacterial strains isolated from phenol-contaminated wastewater showed promising potential for phenol bioremediation in industrial wastewaters.
- The study also sheds light on the bacterial consortium’s ability to activate the key enzymes responsible for phenol degradation under aerobic conditions while exhibiting the simultaneous expression of both ortho- and meta-cleavage pathways.
- The use of DDRT-PCR provided valuable insights into the molecular mechanisms underlying the biodegradation process.
- Differential display reverse transcriptase polymerase chain reaction (DDRT-PCR) allowed for the specific amplification and detection of genes responsible for phenol degradation. The expression levels of these genes determined the extent to which both ortho- and meta-cleavage pathways were activated in response to the presence of phenol.

## Data availability statement

The raw data supporting the conclusions of this article will be made available by the authors, without undue reservation.

## Author contributions

SS: Conceptualization, Data curation, Formal analysis, Investigation, Methodology, Project administration, Resources, Software, Visualization, Writing – original draft, Writing – review & editing. DG: Methodology, Software, Supervision, Validation, Visualization, Writing – original draft, Writing – review & editing. SA: Methodology, Software, Supervision, Visualization, Writing – original draft, Writing – review & editing. NG: Formal analysis, Project administration, Software, Supervision, Visualization, Writing – original draft, Writing – review & editing. ZO: Data curation, Investigation, Methodology, Project administration, Resources, Software, Supervision, Validation, Visualization, Writing – original draft, Writing – review & editing.

## Footnotes

^1^www.ncbi.nih.gov
